# Ultrafast pulse propagation time-domain dynamics in dispersive one-dimensional photonic waveguides

**DOI:** 10.1515/nanoph-2024-0567

**Published:** 2025-01-29

**Authors:** Ahmet Oguz Sakin, Ali Murat Demirtas, Hamza Kurt, Mehmet Unlu

**Affiliations:** Department of Electrical and Electronics Engineering, 52995 TOBB University of Economics and Technology, Ankara 06560, Türkiye; School of Electrical Engineering, Korea Advanced Institute of Science and Technology, Daejeon 34141, Korea

**Keywords:** ultrafast photonics, silicon photonics, dispersive integrated waveguides, time-domain

## Abstract

Ultrafast pulses, particularly those with durations under 100 fs, are crucial in achieving unprecedented precision and control in light–matter interactions. However, conventional on-chip photonic platforms are not inherently designed for ultrafast time-domain operations, posing a significant challenge in achieving essential parameters such as high peak power and high temporal resolution. This challenge is particularly pronounced when propagating through integrated waveguides with nonlinear and high-dispersion profiles. In addressing this challenge, we present a design methodology for ultrafast pulse propagation in dispersive integrated waveguides, specifically focused on enhancing the time-domain characteristics of one-dimensional grating waveguides (1DGWs). The proposed methodology aims to determine the optimal structural parameters for achieving maximum peak power, enhanced temporal resolution, and extended pulse storage duration during ultrafast pulse propagation. To validate this approach, we design and fabricate two specialized 1DGWs on a silicon-on-insulator (SOI) platform. A digital finite impulse response (FIR) model, trained with both transmission and phase measurement data, is employed to obtain ultrafast time-domain characteristics, enabling easy extraction of these results. Our approach achieves a 2.8-fold increase in peak power and reduces pulse broadening by 24 %, resulting in a smaller sacrifice in temporal resolution. These results can possibly pave the way for advanced light–matter interactions within dispersive integrated waveguides.

## 1 Introduction

In the perpetually advancing field of ultrafast photonics, the advent of Photonic Integrated Circuits (PICs) employing femtosecond laser pulses represents a significant technological advancement. This progress has wide-ranging implications across various fields, such as quantum photonics [1], [2], photonic neural networks [3], terahertz waveform generation [4], [5], [6], time-division multiplexers [7], and sensing [8]. As these applications become more widespread, fine-tuning the structural parameters of PICs becomes crucial to meet the specific requirements of ultrafast photonic systems. This is particularly important when utilizing femtosecond-duration pulses, which are further complicated by the considerable increase in pulse intensity distortion and temporal broadening during propagation [9]. The propagation of optical Gaussian pulses in linear dispersive media is modeled by the one-dimensional Schrödinger equation, as shown in Equation (1) [10].

(1) $$E\left(z,t\right)=\;A_{0}\frac{T_{0}}{\sqrt{T_{0}^{2}+i\frac{T_{0}^{2}z}{L_{D}}}}\;\exp\left(-\frac{{\left(t-\frac{z}{v_{g}}\right)}^{2}}{2\left(T_{0}^{2}+i\frac{T_{0}^{2}z}{L_{D}}\right)}\right)$$

Here, *E*(*z*, *t*) represents the output electric field envelope of the pulse, where *A*_0_ denotes the initial amplitude, and *T*_0_ corresponds to the initial pulse width. The dispersion length, represented by $L_{D}=\frac{T_{0}^{2}}{\left|\beta_{2}\right|}$ , is determined by the group velocity dispersion (GVD) parameter *β*_2_, and the pulse’s group velocity is denoted by *v*_*g*_. This equation demonstrates that pulse broadening and the decay of peak intensity are significantly correlated with the dispersion length. When comparing Gaussian pulses with durations of 90 fs and 10 ps, the dispersion length indicates that the broadening and intensity decay experienced by a 10 ps pulse over a distance of 1 m are equivalent to those experienced by a 90 fs pulse over just 81 µm. These limitations for near-zero or linear dispersion media become even more pronounced when ultrafast signals propagate through highly dispersive media. It is also important to note that, although not included in Equation (1), nonlinear effects and the third-order dispersion (TOD) coefficient *β*_3_ notably influence the asymmetry and chirping of femtosecond-duration signals [10], [11].

In recent times, there has been a discernible surge of interest in the engineering of slow light phenomena using dispersive integrated waveguides aimed at extending light–matter interaction, improving optical nonlinearity, and establishing a genuine true-time-delay mechanism [12], [13]. One-dimensional grating waveguides (1DGWs), an archetype of highly dispersive structures, have attracted considerable interest due to their streamlined design and reduced dimensionality. This design simplifies the fabrication process by reducing the etched surface area that interacts with the optical mode [14]. Furthermore, when configured with an appropriate Bloch mode profile, 1DGWs are well-suited for high-density integration, effectively minimizing crosstalk in densely populated chip arrangements [15], [16]. Nonetheless, research endeavors focused on achieving slow light using 1DGWs typically target the generation of a sharp dispersion profile in proximity to the band edge [14], [17]. This approach often restricts bandwidth accommodation due to the time-bandwidth product, resulting in significant pulse distortion and posing a notable limitation in ultrafast photonic applications [18], [19]. Despite these problems, this design method continues to be widely applied to almost all dispersive photonic components. Consequently, a significant challenge persists in achieving optimal ultrafast pulse propagation in dispersive integrated waveguides, particularly for 1DGWs to fully exploit their aforementioned advantages.

In light of these challenges, one prospective approach for mitigating the limitation of 1DGWs entails employing a narrower grating modulation width configuration [20], [21]. This configuration yields a Bloch mode profile with greater symmetry and a substantially lower group index. Nevertheless, it necessitates a greatly extended structural length to achieve the desired light–matter interaction, thereby imposing constraints on densely packed chip layouts. In an alternative approach, an increased bandwidth has been achieved while maintaining fixed group index values by utilizing optimized minuscule unit squares that introduce interruptions in the corrugated grating structure [22], [23]. However, a critical aspect often overlooked in optimizing these minuscule unit squares in 1DGWs is the analysis of temporal domain characteristics. Minor variations in bandwidth increment in these studies, despite their impact on the decay in pulse intensity distortion and pulse broadening, are insufficient to solve ultrafast time-domain problems. This issue stems from an excessive focus on frequency domain optimization. Moreover, imperfections such as nonlinear effects, higher-order dispersion, and variations in temporal coupling coefficients within the transition region are often overlooked. Therefore, the design of structures for time-domain studies, particularly in ultrafast pulse propagation through highly dispersive media, should prioritize parameters that are critical for time-domain applications.

In this paper, we present a design methodology for enhancing time-domain dynamics during ultrafast pulse propagation in dispersive integrated waveguides, focusing on 1DGWs. The method optimizes 1DGW structural parameters using a figure of merit (FoM) that maximizes the product of normalized peak intensity and time delay through ultrafast pulse propagation in highly dispersive media. To validate its effectiveness, we design and fabricate two specialized 1DGW structures: one optimized for high group index and the other for ultrafast time-domain characteristics. For a simplified and accurate extraction of the ultrafast time-domain response, we employ a digital FIR approach trained on fully measured data. The following sections outline the proposed methodology and present the results.

## 2 Structure and design

### 2.1 Determining range of physical dimensions

**Figure 1:**
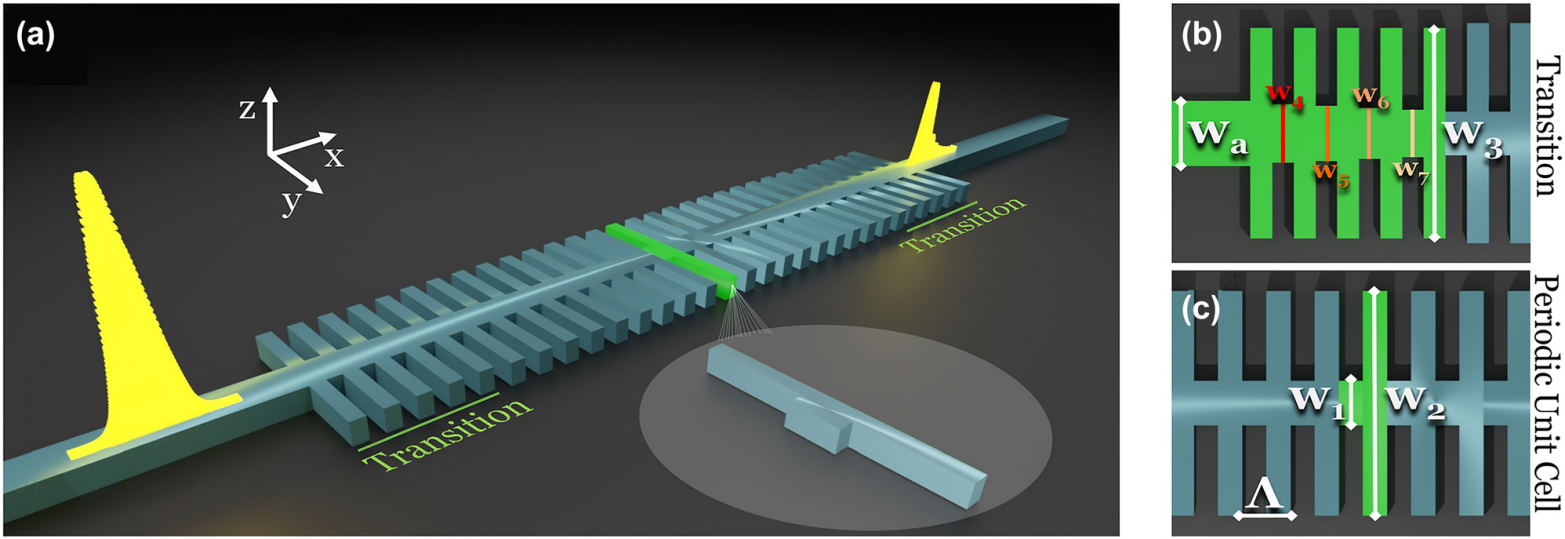
Structural overview of the 1DGW. (a) Schematic representation of the 1DGW structure, (b) top view of the transition region designed for group index smoothing from the strip waveguide to the 1DGW. The labeled dimensions (*w*_a_, *w*_3_, *w*_4_, *w*_5_, *w*_6_, *w*_7_) represent the strip waveguide width, the toothed segment width in the transition region, and gradually narrowing transition widths from *w*_4_ to *w*_7_, respectively, (c) top view of the periodic topology highlighting a single period with the inner waveguide width (*w*_1_), the corrugated grating width (*w*_2_), and the period length (*λ*). These (*w*_1_, *w*_2_, *λ*) are the main parameters for determining the Bragg wavelength of the structure.

Figure 1a–c presents an overview of the 1DGW design. The structural framework is founded upon an SOI substrate, employing a low-resistivity wafer composed of silicon with a depth of 220 nm in conjunction with a buried oxide (BOX) layer measuring 2 μm in thickness. The structure of the 1DGW entails a systematic modulation of the effective refractive index along the optical mode’s propagation axis. The modulation of effective indices within the gratings can be achieved through various sidewall modulation techniques, such as square, sawtooth, triangular, or sinusoidal patterns, etc. [24]. However, these apodization methods introduce undesirable localized variations in the effective refractive index of the inner waveguide and corrugated part of the grating structure. This can lead to phase noise and an increase in output pulse distortion. Hence, we elect to utilize a consistent index modulation denoted by Δ*n* as the configuration of nonapodized gratings.

Due to the Fabry–Pérot-like mechanism within the grating waveguide, Equation (2) describes the phase-matching condition between the transmitted and reflected modes. This equation is highly suitable for theoretically defining the limits of the structural parameters.

(2) $$\lambda_{B}=\left(n_{\text{eff}1}+n_{\text{eff}2}\right)\Lambda$$

Here, *λ*_*B*_ represents the Bragg wavelength, Λ denotes the grating period, *n*_eff1_ and *n*_eff2_ correspond to the effective refractive indices of the inner waveguide (*w*_1_) and the corrugated part (*w*_2_). The parameters Λ, *w*_1_, and *w*_2_ can be seen in the topology of the 1DGW structure, as illustrated in Figure 1c. Increasing the grating modulation width (Δ_mod_ = *w*_2_ − *w*_1_), representing the difference between the inner (*w*_1_) and corrugated (*w*_2_) waveguide widths, enables intentional engineering to achieve a broader operational wavelength range in the reflected mode, which, in forward modes like in this study, corresponds to a reduction in bandwidth [25].

**Figure 2:**
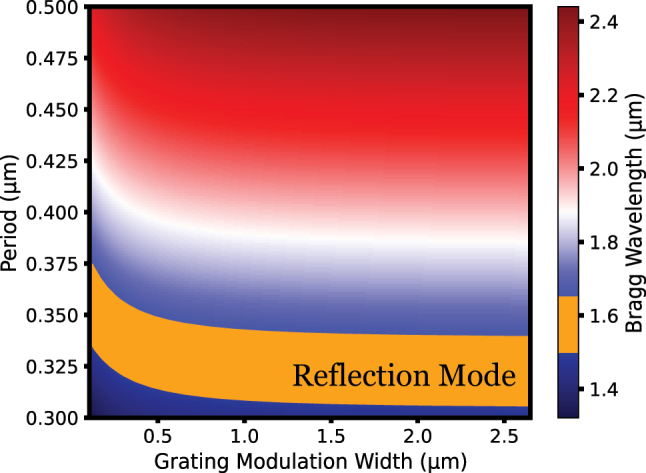
Calculation of the theoretical Bragg wavelength using Equation (2), plotted across a range of grating periods (Λ) and grating modulation width (Δ_mod_ = *w*_2_ − *w*_1_). The segment highlighted in the orange region indicates the parameter space suitable for reflection mode operation within the 1.5–1.65 μm wavelength range.

Therefore, carefully selecting the limits for the parameter range of grating modulation width (Δ_mod_) ensures the inclusion of structures with high bandwidth values in the design process, potentially covering points where temporal signal distortion is minimized. When selecting the maximum and minimum limits of the grating modulation width, it is crucial to define a range that ensures a high coupling coefficient between the inner waveguides and corrugated gratings while enabling a feasible transition design between the strip waveguide and the 1DGW. In light of the challenges associated with determining the minimum and maximum values for the inner waveguide width, as detailed in Supplementary material Section 1, the inner waveguide width (*w*_1_) of the grating was set within the optimally acceptable range, specifically between 0.35 μm and 0.45 μm. When determining the parameter ranges for the corrugated grating width (*w*_2_), and the periodicity (Λ) using Bragg wavelength calculation, the value of *w*_1_ is fixed at 350 nm to satisfy the maximum grating modulation width criterion. The Bragg wavelength is theoretically modeled using Equation (2), across a range of grating periods (Λ) and grating modulation width (Δ_mod_), as outlined in Figure 2. The effective refractive indices (*n*_eff1_ and *n*_eff2_) in Equation (2) are derived from the inner (*w*_1_) and corrugated (*w*_2_) waveguide widths, respectively, using Lumerical MODE. Within the spectrum defined by the Bragg wavelength, light experiences significant reflection. Therefore, to achieve constructive interference within the 1.5–1.65 μm wavelength interval, the corrugated grating width (*w*_2_) and periodicity (Λ) should exceed those corresponding to the reflected regions, as highlighted in the orange areas of Figure 2. This approach helps prevent the emergence of reflection modes. Similarly, choosing higher periodicity values can lead to the operational range of transmission modes shifting to wavelengths exceeding the intended spectral range. Consequently, the corrugated grating and periodicity values should be selected within the ranges of 1.8–2.4 μm and 0.35–0.42 μm, respectively.

The final element to consider in the 1DGW configuration is the design of a high-bandwidth transition zone. Owing to the group-velocity mismatch between the strip waveguide and the 1DGW, there is a noticeable reduction in the butt-coupling efficiency between these structures. Rather than using a linear or adiabatic taper, which is insufficient for supporting slow-light coupling to a higher-band grating waveguide, an anti-Fresnel reflection method-based step taper approach is adopted. This approach enhances energy transfer efficiency, reduces loss, and minimizes oscillations [26]. As illustrated in Figure 1b, the transition region comprises a tapering of the waveguide width through four distinct steps, denoted as *w*_4_, *w*_5_, *w*_6_, and *w*_7_, gradually narrowing from the strip waveguide width (*w*_*a*_) to the inner waveguide width of the 1DGW (*w*_1_). Additionally, within this transition zone, the parameter *w*_3_ – representing the toothed segment – is set to be uniformly 100 nm narrower than the corrugated waveguide widths (*w*_2_) value.

### 2.2 Determining physical dimensions

The system is excited using a Gaussian signal with a full width at half maximum (FWHM) of 90 fs at a wavelength of 1,550 nm. The unit length, comprising mainly 50 periods of the structure, is kept constant to obtain precise pulse storage durations. The analysis is conducted using the three-dimensional finite-difference time-domain (3D-FDTD) solver within the defined parameter ranges. The ultrafast time-domain pulses, obtained at the end of the unit length, are then characterized. To evaluate the characterized time-domain results in relation to peak power, temporal resolution, and pulse storage duration, we employ metrics that directly correspond to these parameters. Specifically, we assess peak electric field (E-field) intensity to represent peak power, pulse broadening to capture the sacrifice of temporal resolution, and time delay to reflect pulse storage duration.

**Figure 3:**
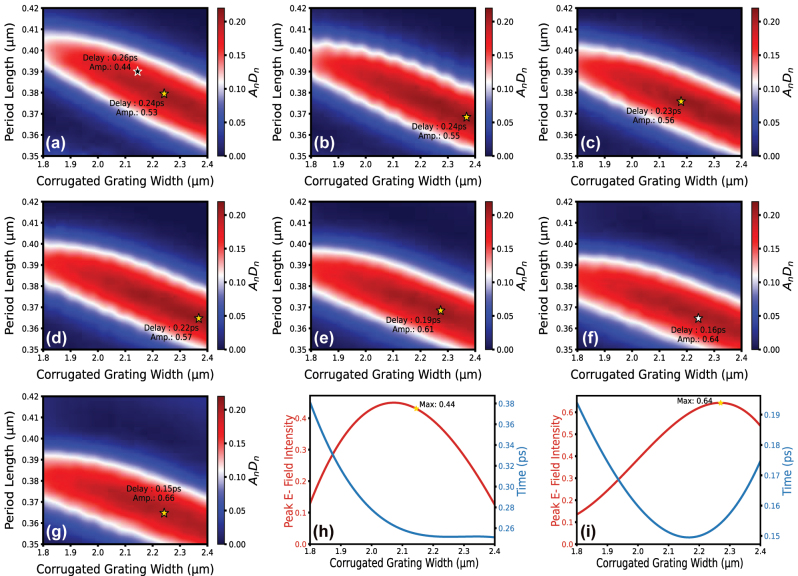
Figure of Merit (FoM), defined as the product of normalized time delay (*D*_*n*_) and normalized peak E-field intensity (*A*_*n*_), is plotted as a function of the corrugated grating width (*x*-axis) and period length (*y*-axis) in subplots (a) to (g). Each subplot corresponds to a different *w*_1_ value: 350 nm (a), 370 nm (b), 390 nm (c), 400 nm (d), 420 nm (e), 450 nm (f), and 460 nm (g). The FoM values are represented by the colorbar. Gold and white stars denote the numerically optimized points that maximize the FoM, while the black star in (a) marks the point where high delay-oriented design parameters are observed. Subplots (h) and (i) show the individual variation of peak E-field intensity (red curve, left axis) and time delay (blue curve, right axis) as functions of the corrugated grating width for fixed *w*_1_ and period length (*λ*) values, corresponding to the black star in (a) and the white star in (f), respectively.

As depicted in Figure 3a–g, the Figure of Merit (FoM), defined as the product of the normalized peak electric field intensity (*A*_*n*_) and the normalized time delay (*D*_*n*_), is plotted to determine the physical parameters required to achieve the best possible time-domain characteristics of 1DGWs. The *x*-axis corresponds to the corrugated grating width (*w*_2_), while the *y*-axis represents the period length (Λ). Across the series of graphs, the inner waveguide width (*w*_1_) is varied from 0.35 to 0.46 μm to evaluate its impact on the FoM. The FoM is calculated for each parameter combination using a time-domain monitor in 3D-FDTD simulations and is visually represented by the color gradient, as indicated by the colorbar labeled *A*_*n*_ *D*_*n*_. It should be noted that the peak electric field intensity and time delay values within the FoM are directly correlated with peak power and pulse storage duration. This design methodology is favored over the traditional approach, which relies on the delay-bandwidth product, particularly for selecting structural parameters based solely on time-domain features. The rationale behind this preference is the directly proportional relationship between the broadband characteristics of the structure and the peak intensity of the output pulse [10]. Consequently, identifying the maximum points of the FoM enables the determination of optimal structural parameters that simultaneously achieve excellent performance in terms of peak power, temporal resolution (implicitly included in the FoM due to the correlation between pulse broadening and peak electric field intensity), and pulse storage duration.

To analyze the structural parameter groups obtained using the FoM, the points corresponding to the best FoM values are marked with gold and white stars in each graph from Figure 3a–g. In Figure 3a, however, the black star represents a high-delay-oriented design, selected using the high time-delay criterion instead of the FoM used in this study. Thus, the design marked with a black star in Figure 3a – representing the traditional high time-delay-oriented design methodology – was selected as a basis for comparison with the proposed time-domain design method. Additionally, to observe the variations in both peak E-field intensity and time delay values among the stars, the term “Amp” next to each star in Figure 3a–g represents the peak E-field intensity value obtained for the corresponding parameter group, while “Delay” indicates the time delay amount for the same parameter group. These values represent actual simulated data obtained using the time monitor in 3D-FDTD, not normalized ones.

Firstly, when examining the gold star in Figure 3a, which corresponds to the peak FoM value for *w*_1_ = 350 nm, the product of *A*_*n*_ *D*_*n*_ is obtained as 0.205. As shown in this figure, the peak E-field intensity and time delay values corresponding to this optimal FoM are 0.53 V/m and 0.24 ps, respectively. In the same graph, when examining the black star, which represents the point corresponding to the high-delay-oriented design, the FoM value is found to be 0.178, while the E-field intensity and time delay values are 0.44 V/m and 0.26 ps, respectively. Here, the trade-off between time delay and E-field intensity is clearly observed. As seen in the transition from the gold star to the black star design, the time delay increases by 8 %, while the E-field intensity decreases by 17 %. This result highlights the importance of time-domain-focused design for dispersive photonic waveguides. It shows that traditional frequency-domain designs optimized for high delay lead to a significant reduction in pulse intensity. Therefore, the trade-off between peak intensity and time delay must be carefully evaluated, particularly for time-domain applications.

When the inner waveguide width (*w*_1_) is increased to 370 nm, the FoM value, shown by the gold star in Figure 3b, increases to 0.212. At this point, the E-field intensity and time delay are observed to be 0.55 V/m and 0.24 ps, respectively, as shown in Figure 3b. Notably, while there is no difference in time delay for the best performance between *w*_1_ = 350 nm and *w*_1_ = 370 nm, the peak E-field intensity increases from 0.53 V/m to 0.55 V/m. This observation further emphasizes the importance of carefully selecting each parameter to achieve optimal ultrafast propagation performance. It demonstrates that the dispersion profile can be adjusted to maintain the same time delay value while increasing the peak E-field intensity.

From Figure 3c–e, when the inner waveguide width (*w*_1_) values are adjusted to 390 nm, 400 nm, and 420 nm, the FoM values are obtained as 0.211, 0.210, and 0.209, respectively. As observed, the changes in FoM are quite minimal, showing a slight decrease of approximately 0.001 as the inner waveguide width increases. However, a noteworthy observation is the decrease in time delay with each increase in width, which correspondingly leads to a gradual rise in peak intensity. When the inner waveguide width (*w*_1_) values are set to 450 nm and 460 nm, the FoM values are obtained as 0.205 and 0.202, respectively. It is also noteworthy that up to *w*_1_ = 450 nm, the FoM value consistently decreases at a rate of 0.001 or 0.002 as shown in Supplementary material Section 2, but it begins to decrease at a higher rate of 0.003 at *w*_1_ = 460 nm. As shown in Supplementary material Section 2, this decreasing trend continues, and at *w*_1_ = 470 nm, 500 nm, and 600 nm, the FoM value further drops to 0.200, 0.195, and 0.180, respectively. The primary reason for this behavior is as follows: as the inner waveguide width (*w*_1_) increases, the corresponding *n*_eff1_ also increases. According to the Bragg condition expressed in Equation (2), this results in a shift in the Bragg wavelength. To maintain high FoM values at 1,550 nm, the applied design methodology must counteract this shift by suppressing the change in Bragg wavelength, ensuring the structure operates in the forward mode. There are two main approaches to achieve this: reducing the corrugated grating width (*w*_2_), which also decreases *n*_eff2_ to balance the increase in *n*_eff1_, or decreasing the period length. When the corrugated grating width (*w*_2_) is reduced, the difference between *n*_eff1_ and *n*_eff2_ diminishes, which significantly reduces the obtained time delay. The second alternative is to reduce the period length to maintain a constant Bragg wavelength. However, as shown in Figure 2, reducing the period length provides a highly limited range before the structure transitions into reflection mode. As shown in Supplementary material Section 2, when *w*_1_ = 600 nm is selected, the period length is nearly reduced to 350 nm, preventing the shift in the Bragg wavelength. However, as seen in Figure 2, further reducing the period length risks transitioning the structure into reflection mode, and it is evident that as *w*_1_ increases, there will be limitations on reducing the period length. When the period length reaches the reflection mode threshold and becomes limited, the only parameter that can compensate for the increase in *w*_1_ is the corrugated width. However, as *n*_eff1_ and *n*_eff2_ values become closer to each other, this ultimately causes a significant reduction in the time delay. Moreover, as *w*_1_ increases extremely and, as a result, the grating modulation width decreases, Figure 2 shows that this eventually causes the structure to operate entirely in the reflection mode. As a result, as *w*_1_ increases, there is a significant decline in FoM-based performance, with the first sharp rate of decrease starting at *w*_1_ = 460 nm, as previously explained. Therefore, within the scope of this study, the acceptable range with an upper limit of 450 nm will continue to be used.

In Figure 3f, the best FoM value is marked by the white star, corresponding to *w*_1_ = 450 nm. At this point, the E-field amplitude is 0.64 V/m and the time delay is 0.16 ps. Considering the best FoM values obtained, the peak value is achieved at *w*_1_ = 370 nm in Figure 3b, with a FoM of 0.212. A closely similar performance range in terms of FoM, referred to as the acceptable range, is observed between 350 nm (FoM: 0.205) and 450 nm (FoM: 0.205). Among these optimal cases, a trade-off exists between the peak E-field intensity and time delay. When comparing the best FoM points in Figure 3b and f, a 33 % decrease in time delay is observed, accompanied by a 16 % increase in the peak E-field intensity. In this trade-off scenario, the optimal approach is to make application-specific design choices. For applications requiring high time delay values, such as tens of picoseconds, the design presented in Figure 3b is more promising. This is because the desired time delay can be achieved with fewer periods, and as will be discussed later, reducing the number of periods also minimizes the decrease in peak E-field intensity. For sub-picosecond applications, as considered in this study, the design obtained in Figure 3f is more promising for achieving higher peak E-field intensity. As will be shown in more detail later, since the desired time delay is lower, the need for a large number of periods is eliminated. Consequently, the design in Figure 3f achieves the desired time delay without reducing the peak E-field intensity to the level seen in the design of Figure 3b. Therefore, within the scope of this study, to highlight the significance of the proposed ultrafast time-domain-oriented design, we select two specific designs: the high-delay-oriented design, marked by a black star in Figure 3a, and the ultrafast time-domain-oriented design, indicated by a white star in Figure 3f. The list of parameters for the structures represented by the black and white stars in Figure 3a and f is provided in Table 1. 1DGW#1 (black star, Figure 3a) corresponds to the design parameters for the high-delay-oriented structure, while 1DGW#2 (white star, Figure 3f) represents the parameters for the ultrafast time-domain-oriented structure.

**Table 1:** List of the parameters for selected 1DGWs.

	1DGW #1	1DGW #2
DC [%]	50	50
*w*_1_ [nm]	350	450
*w*_2_ [nm]	2,150	2,270
*w*_3_ [nm]	2,050	2,170
Λ [nm]	390	365
# Of periods	75, 150	150

DC, Duty Cycle.

Figure 3h and i illustrate how the time delay and peak intensity values vary with respect to the corrugated grating width (*w*_2_) at the period values (Λ) of the specifically chosen designs. It should be noted here that the optimal corrugated width values (*w*_2_) exhibit nearly the same sensitivity on the FoM as the inner waveguide width (*w*_1_). To support this claim, FoM values were analyzed for the selected time-domain-oriented design, showing that when *w*_2_ = 2.2 μm, the FoM was 0.194, and when *w*_2_ = 2.3 μm, it increased to 0.201. Additionally, as the corrugated width (*w*_2_) increases from 2.2 to 2.3 μm, the FoM value shows a continuous rise, reaching its peak at 2.27 μm, and then gradually decreases beyond this point. As previously mentioned, the inner waveguide width value gradually increases, reaches its peak, and then similarly decreases in a gradual manner, following a trend similar to that of the corrugated width. A similar comparison was made for the period length, where the value optimally found at 365 nm was adjusted to 355 nm and 375 nm, resulting in FoM values of 0.129 and 0.155, respectively. Thus, the decreases in the FoM value from the optimal 0.205 point due to ±10 nm variations demonstrate that the period length is the most sensitive parameter.

**Figure 4:**
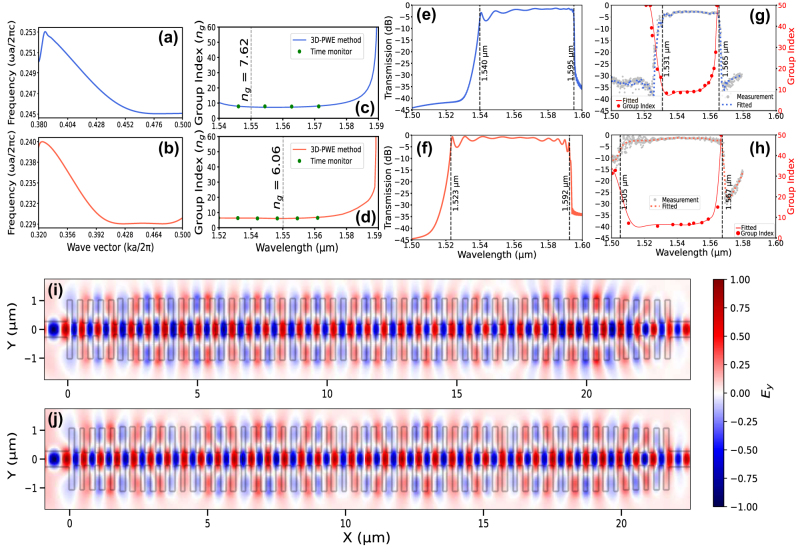
Simulated and measured characteristics of the two specialized 1DGWs. (a)–(b) Dispersion relation of the fundamental quasi-TE Bloch mode for 1DGW#1 (blue) and 1DGW#2 (orange) structures, respectively, (c)–(d) group index results obtained from 3D-PWE and 3D-FDTD simulations for 1DGW#1 (blue) and 1DGW#2 (orange) structures, respectively, (e)–(f) simulated transmission spectra for 1DGW#1 (blue) and 1DGW#2 (orange), with the 3 dB bandwidth boundaries, (g)–(h) measured transmission and group index data for 1DGW#1 (blue) and 1DGW#2 (orange), respectively, (i)–(j) simulated steady-state *E*_*y*_ intensity distributions at 1,550 nm for 1DGW#1 and 1DGW#2, respectively.

Figure 4a and b exhibit the band structures for two selected topologies associated with quasi-transverse electrical (quasi-TE) modes. Their dispersion behavior can be described by the equation $k\left(\omega \right)=n_{B}\left(\omega \right)\frac{\omega }{c}$ . Here, *k*(*ω*) represents the wavevector, *n*_*B*_(*ω*) denotes the Bloch effective index, *ω* is the angular frequency, and *c* is the speed of light in the medium. The group index of structures can be determined from its band structure by employing the formula $n_{g}=\frac{c}{\partial k/\partial \omega }$ , where *n*_*g*_ represents the group index. The three-dimensional plane-wave expansion (3D-PWE) method is utilized to obtain band structures, theoretically enabling the achievement of an infinite group index and local density of states (LDOS) due to the unlimited extent of the simulated geometry [27]. When employing 3D-FDTD simulations with finite geometries, as shown in Figure 4c and d, a notable limitation emerges: beyond a certain threshold, the increased decay rate of the ultrafast pulse makes it impractical to determine the group index from the time monitor [18].

## 3 Device fabrication and experimental verification

### 3.1 Fabrication

**Figure 5:**
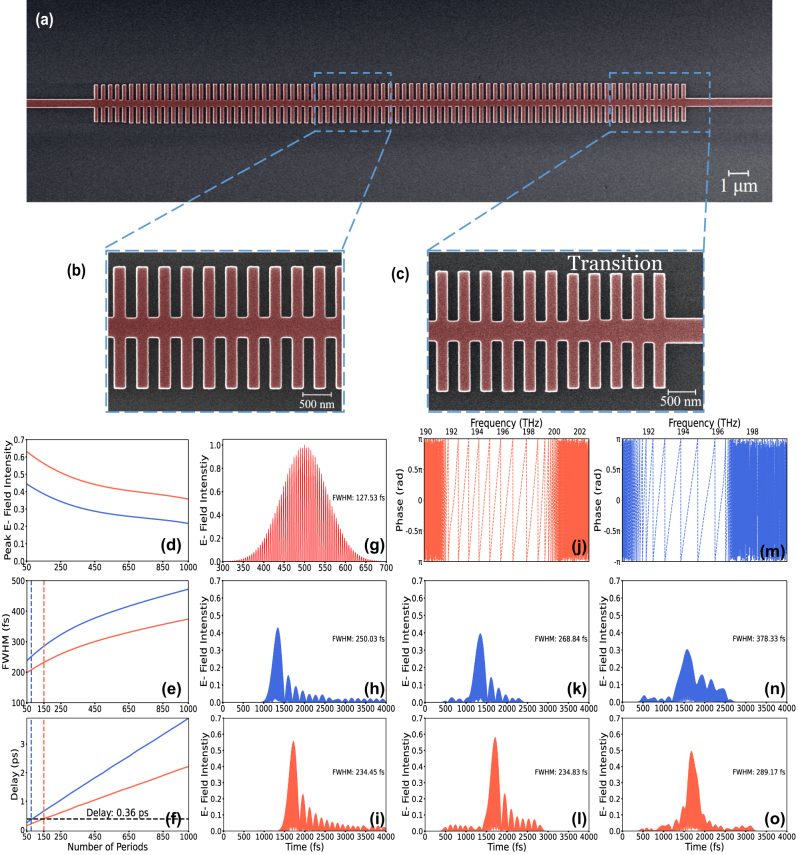
Time-domain dynamics of 1DGWs. (a) Colored SEM image of the 1DGW structure, (b) close-up SEM image of the 1DGW periodic region, (c) close-up SEM image of the transition region, (d) peak E-field intensity variation as a function of the number of periods, (e) FWHM variation as a function of the number of periods, (f) change in time delay as a function of the number of periods, (g) input E-field signal (FWHM value of the power Gaussian signal is 90 fs), (h) simulated time-domain output for the 1DGW#1 structure, (i) simulated time-domain output for the 1DGW#2, (j) phase change as a function of frequency for the 1DGW#2 structure, (k) time-domain output for 1DGW#1 structure using FIR modeling with simulation-based S-parameters, (l) time-domain output for 1DGW#2 structure using FIR modeling with simulation-based S-parameters, (m) phase change as a function of frequency for the 1DGW#1 structure, (n) time-domain output for 1DGW#1 structure using FIR modeling with measurement-based S-parameters, and (o) time-domain output for 1DGW#2 structure using FIR modeling with measurement-based S-parameters.

The designed devices are fabricated using the NanoSOI Multi-Project Wafer (MPW) process by Applied Nanotools Inc., employing direct-write 100 keV electron beam lithography (EBL) [28], [29]. This process utilizes 8-inch SOI wafers with a 220 nm thick device layer and a 2 μm thick buffer oxide layer. The photonic devices are patterned using a JEOL JBX-8100FS EBL system. The designed devices are etched to the buffer oxide layer using an anisotropic inductively coupled plasma-reactive ion etching (ICP-RIE) process. Figure 5a–c showcase a colored scanning electron microscope (SEM) image of the device. After taking the SEM image, a 2.2 μm thick oxide cladding is deposited using a plasma-enhanced chemical vapor deposition (PECVD) process.

### 3.2 Experimental verification

The measurements are conducted using an automated test setup provided by the SiEPIC program test service [28], [29]. Light emitted by a tunable laser (Agilent 81600B), with a wavelength sweeping incrementally from 1,480 to 1,580 nm in 8 pm steps, first passes through a polarization controller to ensure TE polarization. It is then coupled to the input grating coupler via a polarization-maintaining fiber (PMF). The guided light propagates through the 1DGWs and reaches the output grating couplers, where it is collected by a single-mode PMF and subsequently directed into optical power sensors (Agilent 81635A). The transmission measurements include losses associated with the input/output grating couplers, bends, and waveguides. Moreover, the bandwidth of the 1DGWs can only be accurately determined after addressing the bandwidth limitations imposed by the grating couplers. To normalize the transmission values, a baseline measurement is conducted on a strip waveguide equipped with grating couplers and an identical number of bends as the characterized 1DGW systems. The effects outside the device under test (DUT) are eliminated using the average transmission spectra obtained from 10 different loopback structures.

## 4 Results and discussion

### 4.1 Insertion loss and 3 dB bandwidth

The transmission results obtained from simulations for the designed 1DGW structures are depicted in Figure 4e and f, while the corresponding experimental results are illustrated in Figure 4g and h. The simulation results presented in Figure 4e for 1DGW#1 demonstrate that the 3-dB bandwidth is approximately 55 nm and the observed insertion loss at a wavelength of 1.55 μm is 2.6 dB. The 3-dB bandwidth, as determined from measurement, is 34 nm with an insertion loss of 2.65 dB at 1.55 μm, as depicted in Figure 4g. The narrowing of the bandwidth by approximately 21 nm is attributed to fabrication effects, primarily arising from dimensional variations such as sidewall roughness, period, and grating modulation width during the lithography process [30]. As detailed in Section 2, the Bragg wavelength (*λ*_*B*_) is directly related to the grating period (Λ), meaning that even slight deviations in the duty cycle (DC) or period can result in significant shifts in the device’s performance characteristics [31]. Supplementary Section 3 can be referred to for a more detailed analysis of the effects of DC changes. For wavelength drift calculation, the formula Δ*λ* = *λ*_center_measured_ − *λ*_center_simulated_ is employed. With the center frequencies of the 3 dB bandwidths in simulation and measured data calculated as *λ*_center_simulated_ equal to 1,567 nm and *λ*_center_measured_ equal to 1,548 nm, the resulting wavelength drift is 19 nm. This calculation yields a drift wavelength ratio of 1.2 %, highlighting the impact of fabrication variations. In the case of the 1DGW#2 structure, the analysis provides a 3 dB bandwidth of 69 nm from simulations, alongside an insertion loss of 1.13 dB at 1.55 μm as shown in Figure 4f. The insertion loss recorded from measurement results is 1.49 dB at 1.55 μm, within a narrower 3 dB bandwidth of 62 nm, as displayed in Figure 4h. This reflects a decrease of 7 nm in the 3 dB bandwidth from the simulated to the measured results. Using the previously described formula, calculating the wavelength drift ratio results in an approximate drift of 21 nm, equivalent to a drift ratio of 1.4 %. Consequently, according to the measurement results, the time-oriented design exhibits a 44 % lower loss and an 82 % wider 3 dB bandwidth compared to the high group index-based design.

The differentiation of losses can also be elucidated by examining the mode profiles. As illustrated in Figure 4i and j, the spatial distribution of the *E*_*y*_ mode profiles is depicted for the 1DGW#1 and 1DGW#2 structures, respectively. When examining the mode profiles of 1DGW#1 and 1DGW#2, particularly in the transition regions, an increase in scattering is observed for the 1DGW#1 structure, despite both structures having identical transition concepts. This difficulty arises from the challenge of achieving a smooth transition from a high group index dispersion profile (e.g., the 1DGW#1) to a lower group index dispersion profile (e.g., strip waveguide) with a finite transition design, as illustrated in Figure 4c. Despite both structures utilizing the same anti-Fresnel reflection method-based, high-bandwidth transition design, the design of 1DGW#2 demonstrates greater robustness against performance variations in the transition region, which can be significantly affected by fabrication imperfections. This enhanced performance is attributed to the proposed time-domain-oriented design methodology, which incorporates the effects of the transition region.

### 4.2 Group index and slow light bandwidth

Following the transmission results, the group index and slow light bandwidth are demonstrated using simulation data in Figure 4c and d, along with the measurement results in Figure 4g and h. Determining slow light bandwidth (Δ*λ*_SL_) requires identifying the boundaries of the slow light regions. These boundaries are defined as the wavelengths where the group index is within 10 % of the group index measured at a wavelength of 1.55 μm. Conducting a postfabrication analysis of the group index is also essential, as group index values can be significantly affected by imperfections in the transition zone and various other fabrication-related factors. The measured group index for the 1DGWs is determined by analyzing the interference pattern observed in the spectrum of an unbalanced Mach–Zehnder interferometer (MZI) test structure. The first MZI consists of one arm with a 62 μm long grating waveguide and the other arm featuring a 62 μm long, 500 nm wide silicon single-mode strip waveguide, used for calculating the group index of the 1DGW#1 design. Similarly, for the 1DGW#2 design, these lengths change slightly to 58 μm for both arms due to the unit length difference. The group indices of the 1DGW structures are calculated based on the measured transmission results of the MZI structures, as described in Equation (3) [32].

(3) $$n_{g,1DGW}=n_{g,strip}+\frac{\lambda_{\min}\times \lambda_{\max}}{2\times L\times \left(\lambda_{\min}-\lambda_{\max}\right)}$$

Here, *n*_*g*,1*DGW*_ represents the group index of the arm containing the 1DGW, while *n*_*g*,strip_ refers to the group index of the TE mode strip waveguide, which has been experimentally determined to be approximately 4.11. The parameter *L* denotes the physical length of the 1DGW, and *λ*_max_ and *λ*_min_ indicate the wavelengths corresponding to the local power maximum and minimum, respectively.

As illustrated in Figure 4c, for 1DGW#1, the simulated group index *n*_*g*_ at a wavelength of 1.55 μm is determined to be 7.62, with a corresponding slow light bandwidth (Δ*λ*_SL_) of 29 nm, spanning the wavelength range of 1,546–1,575 nm. Similarly, as illustrated in Figure 4d, for 1DGW#2, which is specifically designed for the propagation of ultrafast pulses, the simulated group index is calculated as 6.06, with a Δ*λ*_SL_ of 34 nm, covering a wavelength range from 1,531 to 1,565 nm. Considering the experimental results, Figure 4g and h reveal that the group indices at a wavelength of 1.55 μm are 9.06 and 7.19 for 1DGW#1 and 1DGW#2, respectively. For the measured slow light bandwidth values, it is observed that the slow light bandwidth Δ*λ*_SL_ for the structure 1DGW#1 spans 22 nm, ranging from 1,532 to 1,554 nm. For the structure 1DGW#2, the slow light bandwidth Δ*λ*_SL_ of 21 nm is recorded, extending from 1,536 nm to 1,557 nm. When comparing the measured and simulated results, a decrease of 7 nm in the slow light bandwidth Δ*λ*_SL_ for 1DGW#1 and 13 nm for 1DGW#2 is observed. Changes in the slow light bandwidth and group index are primarily attributed to shifts in the center wavelength and imperfections caused by fabrication processes. These factors significantly contribute to the observed variations in the slow light bandwidth between simulation and experimental data [23]. Despite the wavelength drift and deviations in the slow light region, the overall trend aligns well with the simulated expectations.

### 4.3 Ultrafast pulse propagation time-domain dynamics in 1DGWs

After determining the transmission and group index values, which are essential for obtaining the scattering parameters for the FIR model, we present the time-domain analysis of the designed structure, based on both simulations and experiments, as shown in Figure 5d–o. In all graphs, the blue color represents the results of the 1DGW#1 design, whereas the orange color corresponds to the results of the 1DGW#2 design. The simulation results shown in Figure 5d–i are obtained using the 3D-FDTD time monitor. In these simulations, a Gaussian signal is used as input, defined by a central wavelength of 1,550 nm, a FWHM of 90 fs, and a time offset of 500 fs. As observed in Figure 5d, an increase in the number of periods leads to a great reduction in peak intensity. The proposed 1DGW#2 structure demonstrates a peak intensity value of approximately 0.4 V/m over 1,000 periods, as shown by the orange line. The peak intensity value decreases to approximately 0.2 V/m for the same number of periods in the 1DGW#1 structure, as represented by the blue line. The peak intensity decay rate highlights the advantage of designing the structure with a focus on ultrafast time-domain characteristics.

Upon examining Figure 5e, it becomes apparent that the FWHM value expands as the number of periods increases. The rate at which the FWHM increases for the 1DGW#2 structure is significantly less than that for the 1DGW#1 structure. The lower pulse broadening value observed in the 1DGW#2 structure can be attributed to the correlation between the peak intensity and the pulse broadening value. The trend similarity between Figure 5d and e provides clear evidence supporting this statement. When comparing the FWHM values for 1,000 periods, it is shown that the pulse broadening rate in the 1DGW#2 structure is 26 % lower. However, to ensure a more accurate comparison, the designed structures should be evaluated based on an equal time delay criterion. Figure 5f depicts the correlation between the number of periods and time delay. This visualization highlights the points at which the two specialized designs provide equivalent time delay values. Therefore, the target time delay is established as four times the FWHM of the input signal. It is observed that the 1DGW#1 and 1DGW#2 structures reach this delay at 75 and 150 periods, respectively, resulting in a time delay of 0.36 ps, as indicated by the dashed horizontal line in Figure 5f.

The applied input E-field signal, where the FWHM is observed to be 127 fs, can be seen in Figure 5g (for power, this corresponds to 90 fs). Upon examining the time-domain outputs presented in Figure 5h and i for selected specific time delay, it is observed that the blue pulse, representing the output from 1DGW#1, has a peak intensity value of approximately 0.43 V/m and an FWHM value of 250 fs. Moreover, 1DGW#2, engineered for enhanced performance in the ultrafast time-domain, is depicted in orange, as shown in Figure 5i. The pulse for 1DGW#2 shows a peak E-field intensity value of 0.56 V/m and an FWHM of 234 fs. Notably, the decrease in peak intensity value is significantly minimized during the same time delay value for 1DGW#2. Moreover, the 1DGW#2 structure achieved a 6 % reduction in pulse broadening compared to 1DGW#1, thereby also reducing pulse distortion under the equal time delay condition. As previously noted, the primary focus of this design method is the peak power level and its associated temporal resolution. Therefore, the power-based comparison should be derived using the relationship *I* ∝ *E* ^2^, where *I* is the peak power intensity and *E* is the E-field intensity. The FWHM of the power intensity follows $\Delta t_{I}=\frac{\Delta t_{E}}{\sqrt{2}}$ , where Δ*t*_*I*_ and Δ*t*_*E*_ represent the FWHM of the power and E-field, respectively. We should also note that, in this design method, we use the FWHM of the signals to evaluate and compare their temporal resolution. Consequently, in a power-based comparison, 1DGW#2 demonstrates a 72 % increase in peak power and a 6 % smaller reduction in temporal resolution, as indicated by the decreased FWHM broadening, relative to 1DGW#1.

To obtain the time-domain response of 1DGWs experimentally, the FIR model trained with complex and band-limited scattering parameters obtained from continuous wave-based measurement is employed. This approach is an effective and accurate method for analyzing ultrafast pulse propagation in passive, lossy, linear, and time-invariant (LTI) systems. This is because these structures, such as designed 1DGWs, are characterized by scattering parameters that adeptly account for nonidealities such as higher-order dispersion, wavelength-dependent losses, and inaccuracies in coupling coefficients [33]. Hence, it can be seen that all the necessary information to obtain time-domain response can be acquired from classical optical measurements.

The FIR model is designed as the bandpass system, enabling faster results due to the absence of feedback-related pole residues, unlike in applications based on vector fitting [34], [35]. This modeling employs Lumerical Interconnect, a commercial software [36]. The software’s capability to estimate modeling taps based on group delay, a feature intrinsic to the FIR modeling approach, enabled the realization of modelings with superior performance [37]. When attempting to design with the Lumerical Interconnect IIR model based on the vector fitting algorithm using the same scattering parameters, numerous challenges are encountered, including difficulties in adjusting the parameters concerning the group delay and in determining the number of taps. Consequently, this approach yielded results that are more time-consuming and of lower resolution than those obtained with the FIR model.

To evaluate the high-performance capabilities of the FIR approach, we initially designed an FIR model incorporating the scattering parameters of 1DGW#1 and 1DGW#2, utilizing Lumerical’s 3D-FDTD simulation. The outcomes for 1DGW#1 and 1DGW#2 are depicted in Figure 5k and l, respectively. When Figure 5k is analyzed, the peak E-field intensity is recorded at 0.39 V/m with a 9 % discrepancy from the 3D-FDTD results, and the FWHM is established at 268 fs, showing a 7 % difference compared to the 3D-FDTD findings. Moreover, upon examining Figure 5l, it is observed that the peak intensity achieves a value of 0.58 V/m, indicating a minimal discrepancy of 4 % compared to the 3D-FDTD result. Additionally, the FWHM value is determined to be 234 fs, which closely aligns with the result from the 3D-FDTD analysis. Therefore, it has been demonstrated that the error rates are significantly low.

Following the same approach as explained above, we extract the measured time-domain response of the fabricated structures by employing the FIR modeling to the measured scattering parameters. To determine the phase data, the group delay is first calculated using the formula $\tau_{g}=\frac{L}{n_{g}/c}$ where *τ*_*g*_ is the group delay. Subsequently, by employing Equation (4), the phase is calculated from the group delay, which itself is determined through linear interpolation of the group index points, utilizing the cumulative trapezoidal numerical integration technique [38].

(4) $$\tau_{g}=\frac{\Delta \phi }{\Delta \omega }$$

Here, Δ*ϕ* denotes the change in phase throughout the observed frequency span Δ*w*. Upon examining Figure 5j and m, the phase versus frequency results for 1DGW#2 and 1DGW#1 can be observed, respectively, within the range of −*π* to *π*. Utilizing the acquired phase data and transmission measurements, we derive the scattering parameters necessary for training the FIR model. Figure 5n and o displays the results of ultrafast signals transmitted through 1DGW#1 and 1DGW#2, respectively, using the trained FIR model. When analyzing the output for the 1DGW#1 structure in Figure 5n, it is observed that the FWHM value is 378 fs, and the peak intensity value is approximately 0.3 V/m. When comparing the simulation results with the experimental results, a 30 % decrease in peak intensity and a 51 % increase in FWHM expansion are observed for the 1DGW#1 structure. Upon analyzing Figure 5o for the 1DGW#2 structure, it is observed that the peak intensity value has decreased to 0.50 V/m. The FWHM value of 1DGW#2 is observed to be 289 fs. Hence, for the 1DGW#2 structure, the peak intensity value decreased by 11 %, and the FWHM expansion increased by 24 % compared to the simulation result.

The observed discrepancies between the experimental FIR measurements and simulations primarily arise from fabrication-related imperfections and the differences between the material parameters used in simulations and those of the fabricated structures. Geometric deviations, like the slight rounding of grating edges, are commonly caused by the limited resolution of e-beam lithography, the natural properties of resist development, and challenges in ensuring anisotropic etching during deep reactive ion etching (RIE) processes. As stated in Supplementary material Section 1, even minor changes in low-width values, such as the inner waveguide width, can significantly impact the effective refractive index profile. Consequently, these geometric deviations can have a pronounced effect on the effective refractive index profile, which, considering its influence on the time-domain character, becomes a critical factor to explain the observed discrepancies.

Additionally, deviations in the period length and refractive index inconsistencies between the simulated and fabricated materials further contribute to fabrication imperfections. As noted in Table 1, the period length of the 1DGW#2 structure is 365 nm, and the half-spacing of the grating is 182.5 nm, which is beyond the precision achievable by e-beam lithography. The general fabrication tolerance observed in this process is within the 10–20 nm range, making it evident that precise features such as 182.5 nm cannot be accurately fabricated. A similar situation is expected to be observed for the 1DGW#1 structure, due to its corrugated width value of 2,145 nm. Therefore, the toothed segment (*w*_3_) in the transition structure for 1DGW#1 is selected as 2045 nm. Considering that the transition structure’s characteristics are particularly sensitive to fabrication-induced variations due to increased group index values near the bandgap, even minor deviations in the dimensions can lead to performance degradation.

As previously described, this study demonstrates that the period length is the most sensitive parameter affecting the time-domain character. Additionally, considering the differences in effective refractive index values between simulation and fabrication, these factors explicitly explain the variations observed in the time-domain profiles between simulation and experimental FIR. Moreover, as noted in the previous sections, the observed narrowing of the bandwidth, the wavelength drift ratio values, and the increase in group index values in the fabrication results also support these discrepancies. For 1DGW#1, considering these parameters, a 21 nm band narrowing and a 19 nm wavelength drift are observed, whereas, for 1DGW#2, a 7 nm band narrowing and a 21 nm wavelength drift are obtained. Especially, the limited decrease in bandwidth explains the less pronounced degradation observed in 1DGW#2.

When comparing the experimental results of the two designs, the peak E-field intensity value of the time domain-oriented 1DGW#2 structure is observed to be 66 % higher than that of 1DGW#1. By focusing on power level metrics, based on the previously explained relationship between power and the E-field, the experimental measurements show that 1DGW#2’s peak power intensity is 2.8 times higher than that of 1DGW#1. Furthermore, the broadening in the FWHM value is reduced by 24 % for 1DGW#2 compared to 1DGW#1. In a power signal-based comparison of FWHM, this reduction in broadening is also 24 %. This is a consequence of its higher dispersion profile-based design, which makes it more sensitive to fabrication imperfections. Consequently, the 6 % difference observed in simulations for the FWHM has increased to 24 % in the measurements. In contrast, despite a slight narrowing of the 3 dB bandwidth and minor wavelength drift, the superior performance of the 1DGW#2 structure can be attributed to its dispersion profile, optimized for time-domain operations, which results in more robust performance against fabrication imperfections. Therefore, it exhibits a 24 % smaller sacrifice in temporal resolution, as reflected by the decreased FWHM broadening, in comparison to 1DGW#1.

## 5 Conclusions

This study presents a time-domain dynamics-based design method for dispersive integrated waveguides, with a specific focus on 1DGWs for ultrafast pulse propagation. This study primarily focuses on achieving high peak power and high temporal resolution in dispersive integrated waveguides for femtosecond technology applications. However, the balance between these factors can be adjusted for specific applications using the proposed design methodology. The implications of our proposed design approach are far-reaching, with immediate applications in temporal-mode encoding using highly dispersive structures for high-dimensional quantum information processing [39]. Particularly, in systems utilizing ultrafast pulses to generate diverse quantum states, the reduction of pulse shape distortion becomes paramount for maintaining system fidelity. Furthermore, our study eases the challenge of measuring complex temporal waveforms using the FIR model approach, a long-standing obstacle in high-dimensional quantum information processing. Our approach also holds significant potential for various other applications, including photonic neuromorphic computing, photonic neural networks, photonic LIDAR, and terahertz waveform generation.

## Supplementary Material

Supplementary Material Details
