# Supplementary material for: Ultrafast pulse propagation time-domain dynamics in dispersive one-dimensional photonic waveguides
Source: Nanophotonics. 2025 Jan 29;14(3):353–66. doi: 10.1515/nanoph-2024-0567 (PMC11831391; doi:10.1515/nanoph-2024-0567)
Supplement: Supplementary file 1 — Supplementary Material Details [file j_nanoph-2024-0567_suppl_001.pdf]

Supplementary Materials for

Ultrafast pulse propagation time-domain dynamics in dispersive one-dimensional photonic waveguides

Ahmet Oguz Sakin[1], Ali Murat Demirtas[1], Hamza Kurt[2], Mehmet Unlu[1,*]

[1] *Department of Electrical and Electronics Engineering, TOBB University of Economics and Technology, Ankara, Turkey*

[2] *School of Electrical Engineering, Korea Advanced Institute of Science and Technology, Daejeon, Korea*

[*] *Corresponding author: munlu@etu.edu.tr*

S.1 Effective Refractive Index Analysis for Grating Segments in 1DGWs

One-dimensional grating waveguides (1DGWs) fundamentally operate based on the periodic variation of the effective refractive index along their periodic topology. In such waveguides, Bragg reflection causes light to reflect backward at a specific wavelength range. However, some of the light continues to propagate forward after interacting with the backward-reflected light. This interaction slows down the light significantly, and in this study, a part of the slowed-down light propagates in the forward direction. When the grating modulation width ($\Delta_{\text{mod}} = w_2 - w_1$) is increased, the contrast between the effective refractive indices n_{eff1} and n_{eff2} in adjacent sections of the grating becomes larger. This stronger contrast creates a greater mode perturbation in the structure, making the n_{eff}-vs-λ curve steeper near the band edges. As a result, $\frac{dn_{\text{eff}}}{d\lambda}$ becomes more negative, leading to an increase in the group index, as shown in Equation S1.

$$n_g = n_{\text{eff}} - \lambda \frac{dn_{\text{eff}}}{d\lambda} \tag{S1}$$

Here, n_g represents the group index, n_{eff} is the effective refractive index, λ is the wavelength, and $\frac{dn_{\text{eff}}}{d\lambda}$ represents dispersion. To achieve this increase in grating modulation width, either the inner waveguide width (w_1) of the 1DGW can be reduced, or the length of the corrugated section (w_2) can be increased. However, the optimal range of these parameters is determined based on two considerations: the transition structure and the coupling coefficient value at the unit cell. When analyzing the inner waveguide width (w_1), smaller widths lead to a greater mode spread into the cladding region, significantly lowering the effective refractive index. Even slight increases in the width result in significant changes in the effective refractive index, as illustrated in Figure S1-(a). In this Figure, the effective refractive index of the 1DGW segments is evaluated as a function of width to observe how the rate of change of the effective refractive index varies with width. This analysis further assesses whether the mode profile is predominantly distributed within the cladding or core regions of the waveguide. In regions where a significant portion of the mode propagates in the cladding, i.e., where the effective refractive index changes rapidly, as shown in Figure S1-(a), several challenges arise.

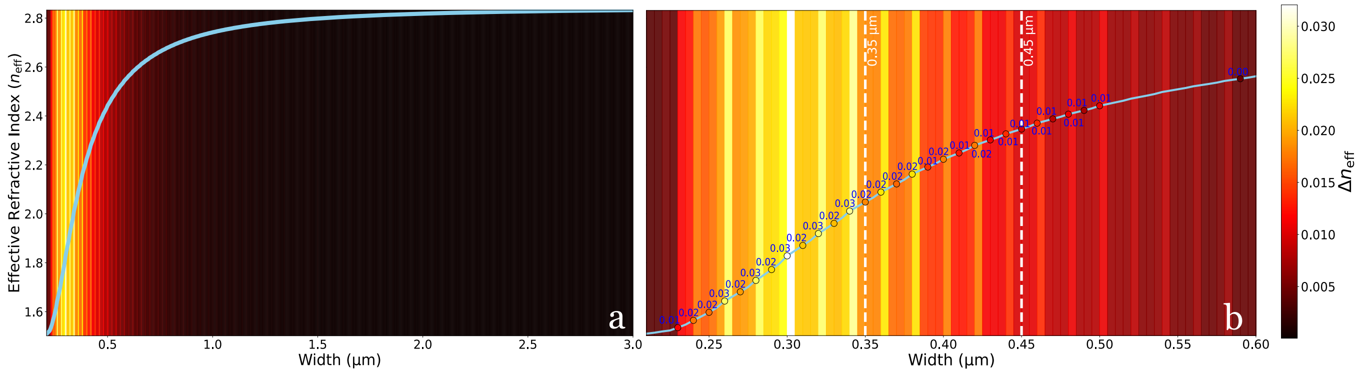

Figure S.1: (a) The variation of the effective refractive index (n_{eff}) is shown as a function of the segment widths. The term segment here refers to the corrugated or inner grating sections. The rate of change is visually represented by a color gradient, as indicated by the colorbar. (b) A magnified view of the critical region, highlighting the acceptable range with specific points annotated to show the change rate of n_{eff}.

Firstly, propagation within the cladding leads to increased optical loss. Furthermore, in transition structures used to couple the strip waveguide mode profile to the Bragg mode profile, the mode mismatch becomes more pronounced, resulting in additional optical loss and a narrowing of the bandwidth. This, in turn, necessitates more complex transition designs. Additionally, given the high fabrication sensitivity of transitions and 1DGWs, modes propagating in the cladding are more susceptible to fabrication imperfections. Therefore, as seen in Figure S1-(b), a change of 0.02 in the effective refractive index has been selected as the baseline, and the point where the change begins to stabilize at 0.02 has been identified as 0.35 μm, which has been chosen as the minimum inner waveguide width (w_1). The maximum limit for the inner waveguide width (w_1) is determined by evaluating its dual effect on pulse characteristics and time delay. As w_1 increases, the grating modulation width decreases, which enhances the forward mode's bandwidth and reduces pulse distortion. However, this narrowing of the grating modulation width simultaneously reduces the effective refractive index contrast (Δn_{eff}) between the inner and corrugated grating regions. This reduction in contrast results in a significant decrease in the achievable time delay. The Figure of Merit (FoM) is determined by multiplying the normalized peak E-field intensity with the normalized time delay, making the optimal point correspond to values where both parameters perform well. In cases where one increases while the other decreases, the FoM value declines accordingly. For this reason, the range where both parameters can deliver optimal results is considered highly suitable for optimization. As shown in Figure S1-(b), when w_1 reaches 0.45 μm, the refractive index variation stabilizes at

approximately 0.01 (For the 430 and 440 nm segments, the effective refractive index change rate was also observed to be 0.01. However, to achieve optimal results in the optimization process, the maximum width was extended up to 0.45 μm.). This point corresponds to the mode becoming largely confined within the core region (or very close to it), where the effective refractive index contrast between the inner and corrugated segments diminishes significantly. Therefore, by selecting 0.45 μm as the maximum limit, the parameter range where significant changes in the effective refractive index (n_{eff}) occur has been included in the optimization process. Thus, the parameter ranges that can exhibit suitable time-domain characteristics have been included in the time-domain optimization process. Figure S2 confirms this approach, as the maximum Figure of Merit (FoM) values are achieved within the defined acceptable range. The peak FoM is observed at w_1 = 370 nm and w_1 = 380 nm, with any deviation from these values, either increasing or decreasing w_1, leading to a reduction in the FoM value.

S.2 Investigation of Structural Parameters of 1DGWs Based on Time-Domain Characteristics

In this section, the time-domain character-based design methodology described in the main manuscript is further detailed by varying the inner waveguide width (w_1) parameter at a smaller resolution to show its impact on the time-domain characteristics more comprehensively. As seen in Figure S2-(a) to (p), the w_1 parameter was varied from 340 nm to 470 nm at 10 nm resolution (Since features under 10 nm are even riskier in e-beam lithography.). Additionally, points at w_1 = 500 nm and w_1 = 600 nm were included to observe the effects of significantly increasing w_1. As stated in Section 1, within the acceptable range of inner waveguide width, the peak FoM values were achieved at w_1 = 370 nm and 380 nm, as shown in Figure S2-(d) and (e), respectively. Decreasing or increasing w_1 beyond these points resulted in a decline in FoM values. This observation supports the parameter range selection, focusing on changes in the effective refractive index.

When examining the peak E-field intensity and time delay values corresponding to the peak FoM, they are found to be 0.55 V/m and 0.24 ps, respectively. Although peak FoM values were achieved at these points, varying w_1 within the range of 350 nm to 450 nm revealed a continuous increase in peak E-field intensity and a decrease in time delay. Considering the similarity in FoM values within this range, all FoM values are viable; however, they present a trade-off between peak intensity and time delay in terms of the time-domain character. As discussed in the main manuscript, this trade-off can be divided into two application-specific cases. For applications requiring time delays exceeding tens of picoseconds (or possibly sub-10 ps), designs with higher unit time delay values, such as those shown in Figure S2-(d) and (e), may be preferred. On the other hand, for this study, which focuses on pulse storage durations based on multiples of the input 90 fs signal's FWHM, the design in Figure S2-(l) was selected. This design exhibits a peak E-field intensity of 0.64 V/m and a time delay of 0.16 ps, achieving the desired 360 fs time delay without reducing the peak E-field intensity to the levels observed in designs with peak FoM values in Figure S2-(d) and (e).

However, another trade-off should be noted here. Designs providing higher time delays require fewer periods, which reduces pulse broadening. While this is not an issue for sub-ps level delays, such as those studied here, it becomes a critical consideration for applications requiring 10 ps or longer delays. In such cases, careful attention must be given to managing pulse broadening.

When the inner waveguide width is set to 600 nm, the obtained time delay is observed to significantly decrease, reaching 0.06 ps. The reason for this is also explained in detail in Supplementary Section 1, where it is described how the acceptable range was selected. Specifically, it highlights that as the grating modulation width decreases, the resulting time delay also starts to decline. As stated in the main manuscript, increasing the corrugation width to prevent a decrease in the grating modulation width requires an increase in the period length to maintain the operational wavelength. However, since the period length is already very close to the reflection mode threshold, its ability to compensate is significantly limited. Therefore, in either case, the situation ultimately results in a reduction in the obtained time delay.

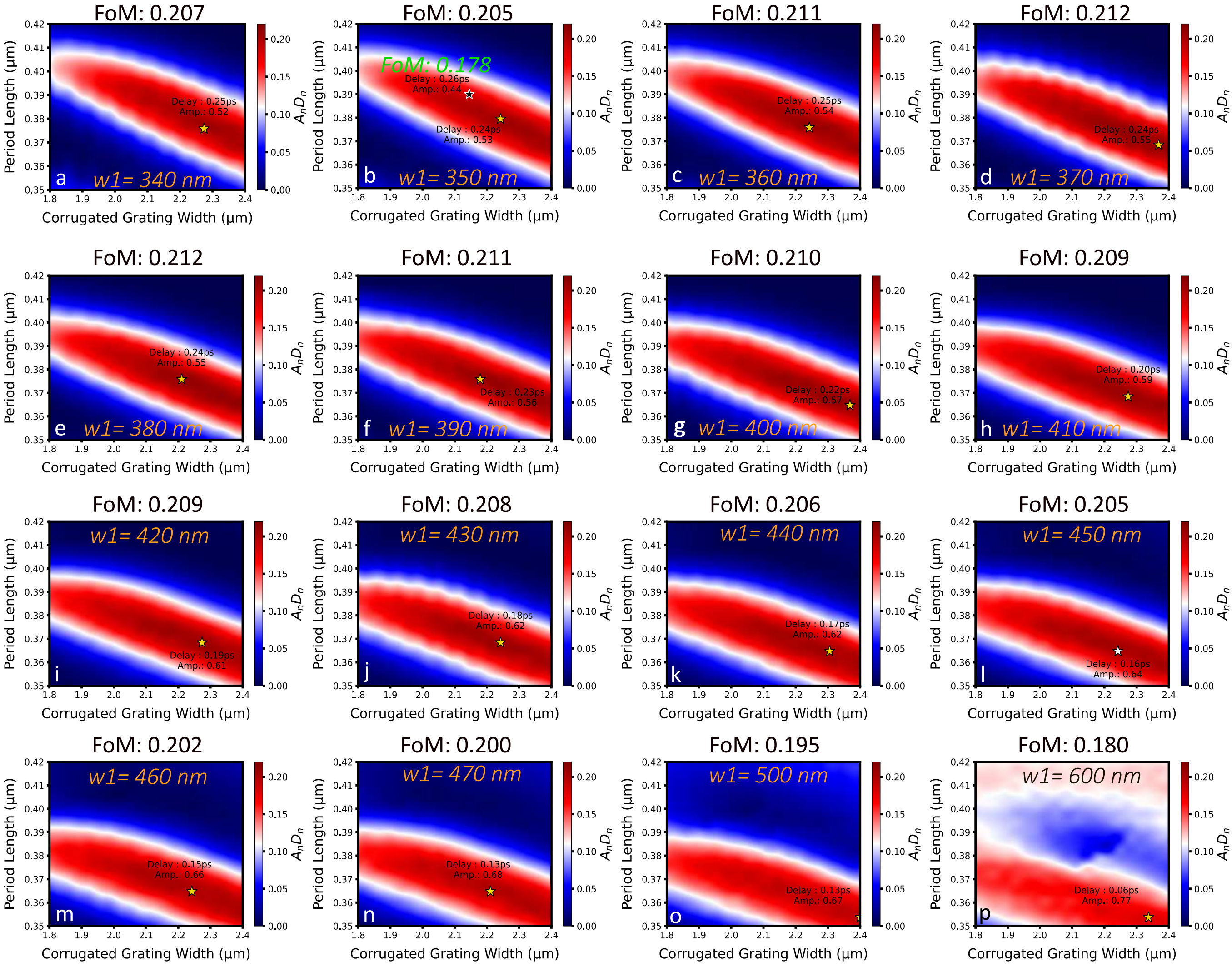

Figure S.2: Figure of Merit (FoM), defined as the product of normalized time delay (D_n) and normalized peak E-field intensity (A_n), is plotted as a function of the corrugated grating width (x-axis) and period length (y-axis) in subplots (a) to (p). Each subplot corresponds to a different w_1 value: 340 nm (a), 350 nm (b), 360 nm (c), 370 nm (d), 380 nm (e), 390 nm (f), 400 nm (g), 410 nm (h), 420 nm (i), 430 nm (j), 440 nm (k), 450 nm (l), 460 nm (m), 470 nm (n), 500 nm (o), and 600 nm (p). The FoM values are represented by the colorbar. Gold and white stars denote the numerically optimized points that maximize the FoM, while the black star in (a) marks the point where high delay-oriented design parameters are observed. The black and white stars represent the fabricated versions used to demonstrate the effectiveness of the time-domain design methodology.

S.3 Investigation of Duty Cycle Effect on Time-Domain Characteristics

The duty cycle (DC) is also one of the key parameters that determine the performance of one-dimensional grating waveguides (1DGWs). DC represents the ratio of the corrugated grating length (L_c) to the total grating period length (Λ). An increase in DC elongates the length of the corrugated segment, which directly affects the Bloch mode's optical path length within each period. As shown in Figure S3, when the period length is evenly distributed as the baseline, increasing the DC results in a decrease in both time delay and peak E-field intensity values. It should be noted that, theoretically, as propagation increases in the corrugated path where n_{eff} is higher, the optical path length would increase, leading to an expected rise in time delay. However, due to the high grating modulation width of the 1DGW structure, as well as additional factors such as transition design and coupling coefficients between gratings, the observed behavior differs. In scenarios where the DC value is integrated into the design process, it will be necessary to develop a more compatible transition design, select an acceptable range suitable for this design, and ensure it aligns with the desired coupling coefficient values. Conversely, when the DC value is reduced, the peak E-field intensity significantly decreases, leading to reflections that create the appearance of an increased time delay.

Moreover, as shown in Figure S3 and stated in the main manuscript, the time-domain-oriented design (1DGW#2) resulted in a period length of 365 nm, making the half spacing for the period equal to 182.5 nm, which is below the resolution limit of e-beam lithography. Consequently, changes in the duty cycle are inevitable. This issue is one of the key reasons for the inconsistency observed between the simulation data and the measurement results of the 1DGW#2 structure.

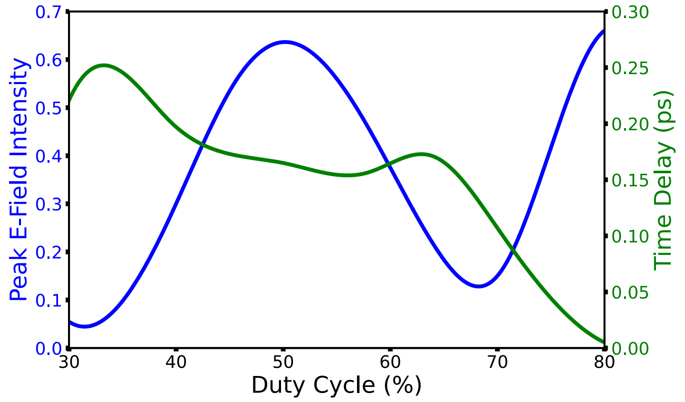

Figure S.3: Dependence of peak E-Field intensity (blue) and time delay (green) on the duty cycle percentage.
